# Differential Protein Expression in the Hemolymph of *Bithynia siamensis goniomphalos* Infected with *Opisthorchis viverrini*

**DOI:** 10.1371/journal.pntd.0005104

**Published:** 2016-11-28

**Authors:** Kulwadee Suwannatrai, Apiporn Suwannatrai, Pairat Tabsripair, Jariya Umka Welbat, Sirikachorn Tangkawattana, Cinzia Cantacessi, Jason Mulvenna, Smarn Tesana, Alex Loukas, Javier Sotillo

**Affiliations:** 1 Food-borne Parasite Research Group, Department of Parasitology, Faculty of Medicine, Khon Kaen University, Khon Kaen, Thailand; 2 Department of Biology, Faculty of Science, Khon Kaen University, Khon Kaen, Thailand; 3 Department of Anatomy, Faculty of Medicine, Khon Kaen University, Khon Kaen, Thailand; 4 Department of Veterinary Pathobiology, Faculty of Veterinary Medicine, Khon Kaen University, Khon Kaen, Thailand; 5 Department of Veterinary Medicine, University of Cambridge, Cambridge, United Kingdom; 6 QIMR-Berghofer Medical Research Institute, Brisbane, Queensland, Australia; 7 Centre for Biodiscovery and Molecular Development of Therapeutics, Australian Institute of Tropical Health and Medicine, James Cook University, Cairns, Queensland, Australia; University of Melbourne, AUSTRALIA

## Abstract

*Bithynia siamensis goniomphalos* is a freshwater snail that serves as the first intermediate host of the human liver fluke *Opisthorchis viverrini*. This parasite is a major public health problem in different countries throughout the Greater Mekong sub-region (Thailand, southern Vietnam, Lao PDR and Cambodia). Chronic *O*. *viverrini* infection also results in a gradual increase of fibrotic tissues in the biliary tract that are associated with hepatobiliary diseases and contribute to cholangiocarcinoma (a fatal type of bile duct cancer). Infectivity of the parasite in the snail host is strongly correlated with destruction of helminths by the snail’s innate immune system, composed of cellular (hemocyte) and humoral (plasma) defense factors. To better understand this important host-parasite interface we applied sequential window acquisition of all theoretical spectra mass spectrometry (SWATH-MS) to identify and quantify the proteins from the hemolymph of *B*. *siamensis goniomphalos* experimentally infected with *O*. *viverrini* and compare them to non-infected snails (control group). A total of 362 and 242 proteins were identified in the hemocytes and plasma, respectively. Of these, 145 and 117 proteins exhibited significant differences in expression upon fluke infection in hemocytes and plasma, respectively. Among the proteins with significantly different expression patterns, we found proteins related to immune response (up-regulated in both hemocyte and plasma of infected snails) and proteins belonging to the structural and motor group (mostly down-regulated in hemocytes but up-regulated in plasma of infected snails). The proteins identified and quantified in this work will provide important information for the understanding of the factors involved in snail defense against *O*. *viverrini* and might facilitate the development of new strategies to control *O*. *viverrini* infection in endemic areas.

## Author Summary

The control of snails involved in transmission of trematodes plays a key role in the fight against these parasitic diseases. While some snails have been studied thoroughly, only few genomic and proteomic studies have focused on *Bithynia siamensis goniomphalos*, the snail intermediate host for *Opisthorchis viverrini*, and none of them separately analyzed the cellular and humoral arms of the snail’s immune system. Here, we applied a label-free proteomic approach to identify and quantify the proteins from the hemolymph of *B*. *siamensis goniomphalos* experimentally infected with *O*. *viverrini*. We identified several immune response proteins that could play critical roles at the snail-trematode interface, generating important new information on the molecular interactions between flukes and their intermediate snail hosts. This work will contribute towards generating a body of knowledge that will ultimately result in the control of *O*. *viverrini* transmission in endemic areas.

## Introduction

*Opisthorchis viverrini* affects over 10 million people in different countries of Greater Mekong sub-region including Thailand, southern Vietnam, Lao PDR and Cambodia [1]. In addition to the problems associated with infection in the definitive human host, chronic inflammation caused by this parasite has been strongly associated with development of cholangiocarcinoma (CCA, a fatal bile duct cancer). Indeed, the International Agency for Research on Cancer (IARC) has recognized *O*. *viverrini* as a group I carcinogen [2, 3]. The complex life cycle of the parasite involves piscivorous mammals (humans, cats and dogs) as definitive hosts, and prosobranch snails of the genus *Bithynia* and freshwater cyprinid fish as first and second intermediate hosts, respectively. Snails become infected by the ingestion of parasite eggs, after which the miracidia hatch from the eggs and, subsequently, penetrate snail tissues developing into sporocysts, which undergo asexual reproduction through the stages of rediae and cercariae. The cercariae exit the snails and penetrate the skin of cyprinid fish hosts to encyst in the muscle and form metacercariae, the infective stage for humans, and which are acquired upon ingestion of undercooked or raw fish [4, 5]. In the endemic regions of northeast Thailand, there is a high prevalence of *O*. *viverrini* infection in humans and fish, although the prevalence of infected *Bithynia* snails is low [6–10]. It has been found that the level of *O*. *viverrini* infection in *Bithynia siamensis goniomphalos* decreases over time, going from 62% infectivity at day 1 to less than 20% at 56 days post infection [11].

The immune system of a snail consists of both humoral and cellular arms. The hemolymph, composed of plasma and hemocytes, not only serves as a source of metabolites and nutrients for parasite development, but also plays a key role in the snail’s response to infections [12–15]. While plasma contains numerous proteins, which may act to directly eliminate pathogens, the hemocytes represent the main immune effectors, playing a major role in the recognition and killing of invading pathogens by different methods such as phagocytosis, encapsulation, and cytotoxic reactions [16–18]. The processes involved in the snail defense mechanisms have been extensively studied in *Biomphalaria* spp. snails [19–22]. For instance, infections with the trematode *Schistosoma mansoni* have been shown to alter the protein levels in both the hemolymph and body of the snail [23–25]. Several proteins whose concentrations change in hemolymph have been identified [26–29]. Different lectins such as selectin, galectin, C-type lectin, and fibrinogen-related proteins (FREPs) were over-expressed after infection and are considered to play an important role in pathogen recognition mechanisms capable of agglutination and binding to intruders [26, 30–34]. In addition, aldolase and myosin have been identified as proteins that regulate hemocyte migration and can be implicated in pathogen killing by a cytotoxic reaction and phagocytosis [28, 29]. Recent studies on *B*. *siamensis goniomphalos* have used different “omics” approaches to study the alterations that occur within defined snail tissues after *O*. *viverrini* infection, such as body and head-foot [29, 35–37], including high-throughput sequencing technologies and protein expression analysis, however molecular changes of the immune effectors of *B*. *siamensis goniomphalos* have yet to be analysed.

In the present study, we applied state-of-the-art mass spectrometry methodologies such as sequential window acquisition of all theoretical spectra (SWATH-MS) to quantify the protein profiles in the hemolymph of *B*. *siamensis goniomphalos* experimentally infected with *O*. *viverrini*. SWATH-MS, a novel technique in mass spectrometry, has recently shown excellent quantitation precision [38]. This technique combines the advantage of precise quantitative accuracy of traditional shotgun proteomics and reproducibility of targeted data analysis similar to that obtained with selected reaction monitoring (SRM) [39–41]. The SWATH-MS technique is performed in two separate steps. The first step (called label-free shotgun or data dependent acquisition (DDA) involves a label-free shotgun approach to create a library of precursors and transitions that are used in a second step (called data independent acquisition –DIA) to quantify proteins in a selected reaction monitoring (SRM) approach from any other sample [38]. In addition, this technique has been used for the identification and proteome-wide quantification of the different proteins in a complex protein sample [38, 41]. While the use of labeled quantitative proteomics techniques such as iTRAQ in *Bythinia*-*Opisthorchis* interactions has proven to be valuable [29], the SWATH-MS technique is a compelling alternative to iTRAQ for protein quantitation of complex mixtures [42]. By characterizing the differential protein expression in the hemolymph of fluke-infected *B*. *siamensis goniomphalos* we have shed light on the molecular basis of the early stages of snail-parasite interactions, and provided information that might lead to the development of novel intervention tools to control parasite transmission by targeting the snail host stage.

## Methods

### Ethics statement

All the protocols used for animal experimentation were approved by the Animal Ethics Committee of Khon Kaen University, based on the ethics of animal experimentation of the National Research Council of Thailand (Ethics clearance number AEKKU51/2557).

### Sample preparation and snail infection

*B*. *siamensis goniomphalos* snails were collected from natural water bodies in the Muang district, Khon Kaen Province (Thailand) and maintained in plastic containers containing de-chlorinated tap water with soil base and provided with boiled gourd leaves and artificial snail food as described previously [43]. Snails were examined for cercarial shedding and re-examined once a week for 8 weeks to ensure they were free of trematode infection. A total of 1,200 snails were used in this experiment.

Embryonated eggs of *O*. *viverrini* were obtained from the faeces of naturally infected cats by sedimentation technique [44]. Briefly, the faeces were mixed with PBS and strained though meshes of different sizes. Samples were sedimented and washed several times with PBS until clear supernatant was obtained, and were kept at room temperature (28±3°C) for 1 week to enable full development. Prior to experimental infection, the eggs were examined under light microscopy to determine full maturation by detecting active movement of the miracidium within the egg shell [45].

The snails (N = 900) were exposed to *O*. *viverrini* mature eggs according to previously described procedures [11]. Three replicates (100 snails per replicate) were used for each of the time points of the experimental infection. Briefly, infection was performed by placing each snail individually with 50 embryonated *O*. *viverrini* eggs into plastic containers containing 5 ml of de-chlorinated tap water for 24h. Uninfected snails were treated in a similar way but no eggs were present in the containers. After infection, the snails were maintained in plastic 50 L containers (38 cm wide x 52 cm long x 31 cm high) at 100 snails per container in de-chlorinated tap water to a height of 10 cm with soil base. Snails were provided with boiled gourd leaves and artificial snail food as described previously [43]. One hundred individual snails were randomly collected at 24, 48 and 96 h post-infection (hpi), and uninfected snails (N = 300) were used as controls. Snail infection was determined by examination of egg hatching in snail faeces (as determined by opening of the operculum) [45]. In addition, after collection of hemolymph, whole soft bodies of all snails were subsequently employed to screen for trematode infection by PCR designed to amplify the ITS regions as described elsewhere [46]. PCR products were amplified and visualized on a 3% agarose gel stained with ethidium bromide. The presence of bands of approximately 380 bp in size was considered diagnostic for *O*. *viverrini* infection.

### Hemolymph collection

Prior to hemolymph collection, the snail shell was cleaned with 70% ethyl alcohol and snails were then soaked for 20 min in phosphate buffer saline (PBS) containing 100 μg/ml of streptomycin and 200 units/ml of penicillin to eliminate potential microorganism contamination. Hemolymph samples were collected by stimulation of the head-foot region of the snail as described previously [12]. Briefly, the head-foot was punctured with a needle resulting in the retraction of the head-foot into the shell and subsequent leakage of hemolymph. The hemolymph was collected using a micropipette and transferred to a new tube on ice to avoid hemocyte aggregation. The hemolymph samples from 100 snails of each replicate and time point were pooled, centrifuged at 3,500 *g* for 10 min at 4°C to pellet hemocytes, and the resulting plasma (supernatant) was transferred to a new centrifuge tube and supplemented with EDTA-Free SigmaFAST Protease inhibitor cocktail tablets (Sigma) as per manufacturer’s instructions. Pellet containing the hemocytes was washed twice with cold PBS, centrifuged at 3,500 *g* for 3 min at 4°C. Plasma and hemocytes were kept at -80°C until processed. Whole soft bodies of all snails were subsequently examined for infection by PCR [46].

### Sample preparation for SWATH-MS analysis

Cell pellet (hemocytes) from each time point was resuspended with 200 μl of lysis buffer containing 3 M Urea, 0.5% SDS, 1% Triton X-100, Protease inhibitor cocktail (EDTA- free), 50 mM Tris-Hcl and 0.5 mM MgCl_2_ and homogenized on ice by sonication (Qsonica, Newtown, CT; 10 cycles of 10 s pulses) followed by incubation on a tube agitator at 4°C for 30 min. Samples were subsequently centrifuged at 12,000 *g* and left at 4°C for 20 min. The pellet was discarded and protein supernatant was concentrated using 10 kDa Amicon filters (Millipore). Plasma proteins were directly concentrated using the same filters. Protein concentrations were determined using a bicinchoninic acid (BCA) protein assay kit (Thermo Fischer, Waltham, USA). The concentrated protein was then stored at -80°C until use. Twenty μg of protein was applied onto a denaturing polyacrylamide gel and subjected to electrophoresis. Protein reduction, alkylation and *in gel* digestion were performed as described previously [47]. Briefly, protein samples were reduced by addition of 0.5 M dithiothreitol at 65°C for 1 h, and then cysteine residues were alkylated with 0.5 M iodoacetamide (Sigma-Aldrich, St. Louis, MO) in darkness at 37°C for 45 min. Proteins were digested using trypsin (Sigma–Aldrich, St. Louis, MO) at 37°C overnight. Prior to SWATH analysis, trypsin-digested samples from each time point were pooled and peptide amount normalized to 5 μg using a C18 Zip-Tip (Merck Millipore, Billerica, MA) according to the manufacturer's protocol, and 1 μL of iRT Kit (Biognosys AG, Schlieren, Switzerland) was added in order to normalize retention times.

### Mass spectrometry

For spectral library generation, samples were analysed by LC-MS/MS on a Shimadzu Prominance Nano HPLC coupled with a SWATH-MS-enabled AB SCIEX Triple TOF 5600+ mass spectrometer. Sample was injected onto a 50 mm 300 μm C18 trap column (Agilent Technologies, Santa Clara, USA) and peptides eluted onto an analytical nano HPLC column (150 mm x 75 μm 300SBC18, 3.5 μm, Agilent Technologies) at a flow rate of 300 nL/min. A gradient of 1–40% buffer B over 35 min followed by a steeper gradient from 40%-80% buffer B over 5 min was used. Buffer B consisted of 90/10 acetonitrile/0.1% formic acid, and buffer A contained 0.1% formic acid (aq). The mass spectrometer was operated in DDA top20 mode, with 500 and 150 ms acquisition times for the MS1 and MS2 scans respectively, and 20 s dynamic exclusion. Rolling collision energy with a collision energy spread of 15 eV was used for fragmentation.

For SWATH-MS data acquisition, the same mass spectrometer and LC-MS/MS setup was operated essentially as described before [39, 40], using a dwell time of 100 ms to cover the mass range of 400–1,200 *m/z* and 32 windows of 25 Da effective isolation width. The collision energy for each window was set using the collision energy of a 2+ ion centered in the middle of the window with a spread of 15 eV.

### Generating the reference spectral library

A comprehensive high-quality spectral library was built following established and validated bioinformatics protocols [48]. All mass TripleTOF 5600+ fragment ion spectra files (.*wiff*) from shotgun data acquisition (DDA mode) were converted into an open format (mzML) using qtofpeakpicker (ProteoWizard, v/3.0.9576) and fragment ion spectrum complexity was subsequently reduced to keep the top 150 peaks using msconvert (ProteoWizard, v/3.0.9576). All datasets were conducted through sequence database searches against a *B*. *siamensis goniomphalos* transcriptome [36] appended with the iRT peptides, a common contaminant database (http://www.thegpm.org/crap/) and decoy sequences using the Trans-Proteomic Pipeline (TPP) software (v/4.8.0), and assigned using parallel searches with X!tandem and Comet. The identified peptides were statistically scored using PeptideProphet and iProphet. False discovery rate (FDR) control was conducted using MAYU, and only proteins with a minimum of two unique peptides per protein and a FDR <1% were used in subsequent analyses. The validated spectra were used to generate a consensus library and all retention times were normalized using SpectraST. To generate a SWATH assay library from the consensus spectra, spectral libraries were formatted for Skyline and the most abundant y and b fragment ions for each spectra with *m*/*z* range 350–1,000 *m*/*z* were selected using a custom Python script as described previously [48].

### SWATH-MS targeted data extraction

Skyline was used for extraction of SWATH-MS data and peptide quantification [49]. Spectral libraries and raw files (.*wiff*) from SWATH-MS experiments were directly imported into Skyline. All possible precursor and fragment ions used for quantitation were selected automatically from the library based on only unique identified peptides for a given protein, typically b- and y-ions, as well as peptides with modifications (Carbamidomethylation and Oxidation). Peptides matching the common contaminant database and decoy sequences were removed from the data set before analysis.

### Bioinformatics analysis

A linear mixed effects model implemented in the open source R package, MSstats (v/3.3.11) was employed to evaluate protein abundance, and only proteins with a *P*-value <0.05 were taken into consideration for further analyses. The gene ontology (GO) terms of proteins with a significant differential expression pattern were studied using Blast2Go [50] and a protein family (Pfam) analysis was performed using HMMER [51]. The enrichment in annotations was calculated for every GO annotation in the three ontologies according to biological processes and molecular functions. Bar charts were generated using different levels of the GO hierarchy and then plotted using the WEGO tool [52]. Heatmaps representing the differentially expressed proteins were generated in R using ggplot2 and clustering was performed using Euclidean distances. The mass spectrometry proteomics data have been deposited in the ProteomeXchange Consortium (http://proteomecentral.proteomexchange.org) via the PRIDE partner repository [53] with the dataset identifier PXD004121.

## Results

**Fig 1:**
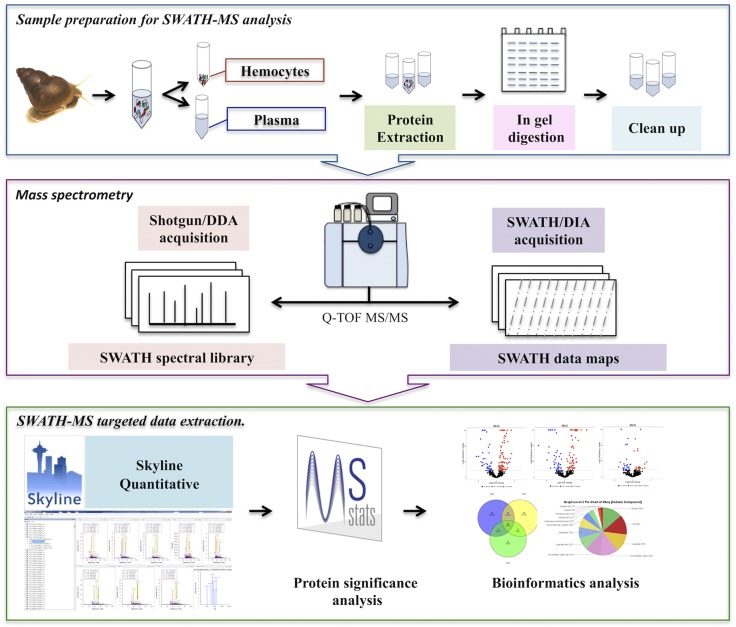
Protocol workflow. Schematic workflow of the protocol followed for the identification and quantification of proteins in the hemolymph of *Opisthorchis viverrini*-infected *Bithynia siamensis goniomphalos* snails.

**Fig 2:**
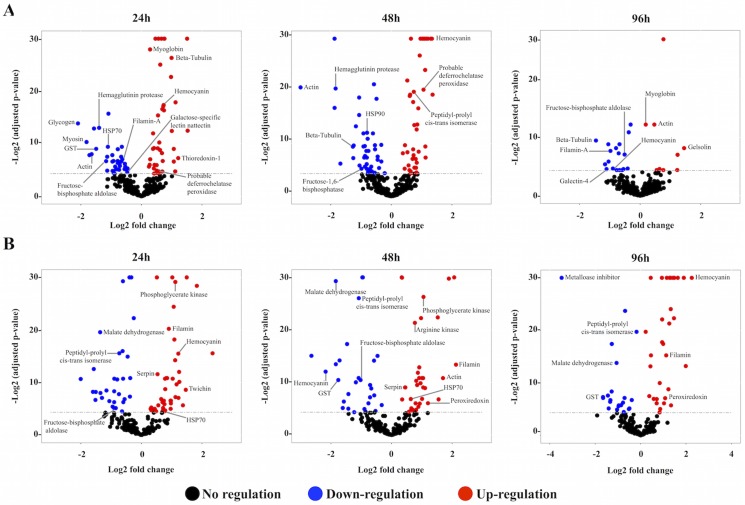
Volcano plot of the significantly differentially expressed proteins. Differentially expressed proteins in hemocytes (A) and plasma (B) of *Bithynia siamensis goniomphalos* following infection with *Opisthorchis viverrini* at different time points (24, 48 and 96 hpi) compared to controls. Volcano plots showing log_2_-fold changes plotted against log_2_ *P*-values. Blue dots represent proteins with significantly down-regulated expression, while red dots represent proteins with significantly up-regulated expression. Proteins of interest discussed in the text have been labelled.

The proteomic alterations in the hemolymph of *B*. *siamensis goniomphalos* infected with *O*. *viverrini* were characterized using the label-free quantitation methodology SWATH-MS (Fig 1). All examined snails were screened individually to determine trematode infection by using cercarial shedding and confirmed by PCR. All snails that were negative by PCR were negative for *O*. *viverrini* and other trematode infections, while PCR-positive snails were visually positive for *O*. *viverrini* (S1 Fig). All challenged snails used for the proteomics analysis were positive by PCR for *O*. *viverrini* infection, while control snails were negative. Samples were prepared for SWATH-MS analysis and, subsequently, subjected to SWATH-MS to generate the spectral library and quantification. Three different biological replicates from each time point were analyzed and a total of 498 and 399 peptides corresponding to 362 and 242 proteins were used to build the hemocyte and plasma libraries respectively; of these, approximately 107 proteins were common to both samples (Table 1). Only proteins with 2 or more identified unique peptides and <1% FDR were selected for the library, although not all the peptides were used for library construction (e.g. peptides with less than 6 transitions). The total number of proteins used in the library corresponded to 0.47% and 0.31% (for hemocytes and plasma respectively) of the whole snail predicted proteome published previously [35]. After importing the SWATH-MS target data into Skyline, we confidently identified a total of 343 and 217 proteins at an FDR <1%, in hemocytes and plasma, respectively. Only peptides found in all 4 time points and all 3 replicates were used for statistical and quantification analysis (S1 and S2 Tables). Statistical analysis of the expression values of proteins from infected versus control snails was conducted, and only proteins showing a *P*-value < 0.05 were considered as significant for further analysis (S3 and S4 Tables). The expression profile (volcano plot) of the identified proteins at different times post-infection is shown in Fig 2. A total of 145 and 117 proteins from hemocytes and plasma, respectively, exhibited significant differences in their expression levels upon infection, of which 36 proteins were common to both samples (Table 1; S5 and S6 Tables).

**Table 1:** Summary results from SWATH-MS analysis. The number of proteins, peptides, precursors and transitions in spectral reference library, filtered at protein FDR 1% and proteins in SWATH-MS datasets are reported. Proteins with statistically significantly differential expression are shown in the bottom half of the table, including the non-redundant total of statistically significant differentially expressed proteins from all time points, as well as numbers of proteins up-regulated and down-regulated within each time point (24, 48 and 96 hpi). Analyses were carried out using MS Stats.

Sample			Hemocytes	Plasma
**Spectral Library**	*Proteins*		362	242
	*Peptides*		498	399
	*Precursors*		528	405
	*Transitions*		3168	2178
**SWATH-MS targeted**	*Proteins*		343	217
	*Peptides*		479	344
**Differentially Expressed Proteins**	*Total differentially expressed proteins*		145	117
	24h	*All*	79	73
		*Up-regulated*	44	42
		*Down-regulated*	35	31
	48h	*All*	86	63
		*Up-regulated*	43	35
		*Down-regulated*	43	28
	96h	*All*	28	54
		*Up-regulated*	10	33
		*Down-regulated*	18	21

**Fig 3:**
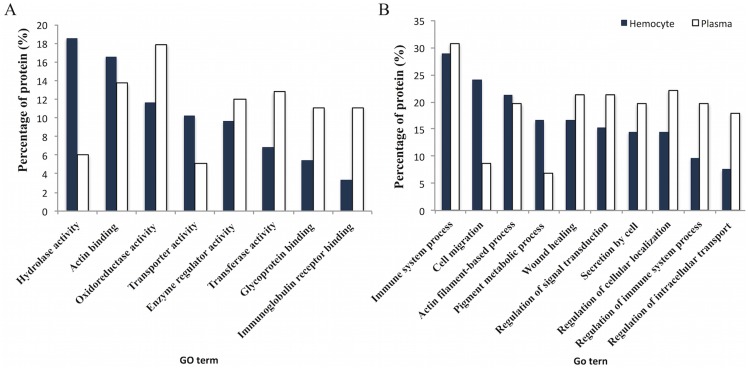
Gene Ontology analysis. A Gene Ontology analysis showing the significantly differentially expressed proteins was performed using Blast2Go [50]. The most abundant gene ontology terms “molecular function” (A) and “biological process” (B) from hemocytes (closed bars) and plasma (open bars) of *Opisthorchis viverrini*-infected *Bithynia siamensis goniomphalos* snails have been plotted.

A Gene Ontology analysis was performed on the significantly differentially expressed proteins from hemocytes and plasma using Blast2GO. The most abundant GO terms within the “molecular function” ontology in hemocytes were assigned as “hydrolase”, “actin-binding” and “oxidoreductase activity”, while the terms related to “oxidoreductase” and “actin-binding activity” together with “enzyme regulator” “and transferase activity” were prominent terms in plasma (Fig 3A). The most abundant GO term in both hemocytes and plasma within “biological process” was “immune system process”. Furthermore, “cell migration” and “actin filament-based process” were prominent terms in hemocytes whereas terms related to “regulation of cellular localization”, “wound healing” and “regulation of signal transduction” were also highly represented terms in plasma (Fig 3B).

**Fig 4:**
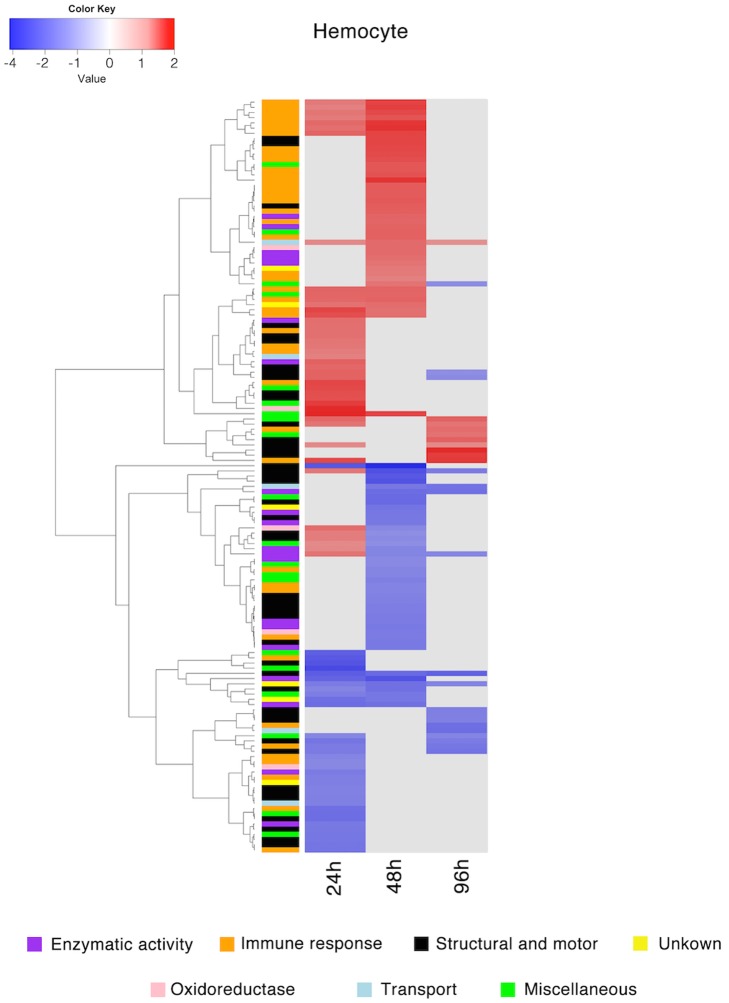
Clustered heatmap of the proteins with a significantly regulated expression profile in the hemocytes from *Bithynia siamensis goniomphalos* snails after infection with *Opisthorchis viverrini*. The relative expression of hemocyte proteins at 24, 48 and 96 hpi of infected snails was compared against the control group (uninfected snails). Proteins were grouped into 7 different categories based on GO annotation and clustering was performed using Euclidean distances based on the expression patterns. Color intensity reflects the corresponding log_2_ fold change and significant up-regulation shown in red, significant down-regulation is shown in blue, and no statistically significant change in abundance is shown in gray.

**Fig 5:**
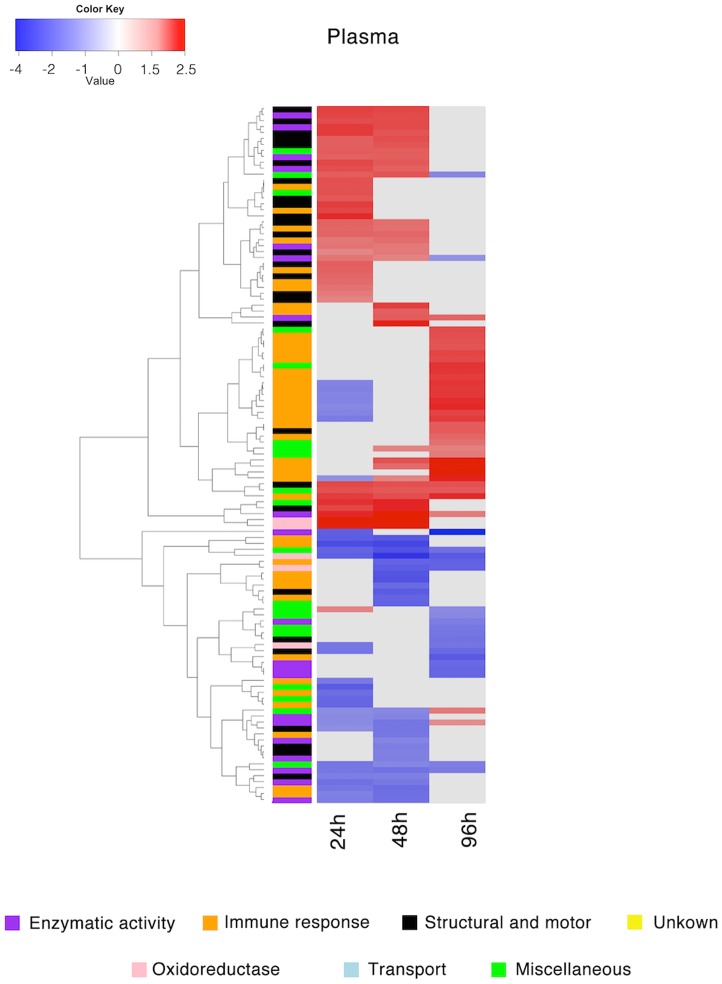
Clustered heatmap of proteins with a significantly regulated expression profile in the plasma of *Bithynia siamensis goniomphalos* snails after infection with *Opisthorchis viverrini*. The relative expression of plasma proteins at 24, 48 and 96 hpi of infected snails were compared against the control group (uninfected snails). Proteins were grouped into 7 different categories based on GO annotation and clustering was performed using Euclidean distances based on the expression patterns. Color intensity reflects the corresponding log_2_ fold change and significant up-regulation shown in red, significant down-regulation is shown in blue, and no statistically significant change in abundance is shown in gray.

Hemocyte proteins with significantly different expression patterns were categorized into the following functional groups: 1) enzymatic activity, 2) immune response, 3) structural and motor, 4) oxidoreductase, 5) transport, 6) miscellaneous and 7) unknown, and plotted in a hierarchical clustered heatmap (Fig 4). The highest number of proteins with a significantly differential expression pattern was found at 48 hpi (86 proteins), followed by 24 hpi (79 proteins). Interestingly, the expression of only 28 proteins was significantly regulated at 96 hpi (S2 Fig). The majority of proteins assigned to immune response had an up-regulated expression pattern, particularly proteins related to hemocyanin, while proteins belonging to the structural and motor group were mostly down-regulated (Fig 4). Furthermore, the expression of several proteins assigned to enzymatic and oxidoreductase activity was up-regulated at 24 hpi but down-regulated at 48 hpi (Fig 4). Similarly, plasma proteins with a significantly regulated expression profile were grouped into the same annotation categories and clustered into a heatmap (Fig 5). The highest number of proteins with a significantly different expression pattern was found at 24 hpi (73 proteins), followed by 48 hpi (63 proteins) and 96 hpi (54 proteins), respectively (S2 Fig). The expression of proteins with an immune response profile and structural and motor proteins was generally up-regulated in the plasma of infected snails, whereas the expression of proteins with an enzymatic activity was significantly down-regulated after *O*. *viverrini* infection (Fig 5).

## Discussion

The digenetic trematode, *O*. *viverrini* has a complex life cycle with freshwater *Bithynia* spp. snails acting as intermediate hosts. Snail immune responses towards trematode infection are known to rely on both plasma and cellular host factors, which are likely to dictate the success or failure of parasite infection [12, 13]. In recent years, anti-pathogen snail host responses have been studied to obtain new information to understand the mechanisms of compatibility between *B*. *siamensis goniomphalos* and *O*. *viverrini* [29, 35–37], although none of these studies have focused on the snail’s immune effector arms.

In this study, we investigated the humoral and cellular responses of the freshwater snail *B*. *siamensis goniomphalos* following exposure to *O*. *viverrini* using a SWATH-MS proteomic approach. We compared the protein profiles in hemocytes and plasma of infected snails at 24, 48 and 96 hpi as early stages of development of parasite versus non-infected snails (control), since circulating hemocytes, in cooperation with humoral factors (plasma), are known to play key roles in the immune response against infection [13, 15, 54]. Hemocytes are phagocytic cells that can recognize and adhere to the surface of parasites, activate cellular cytotoxicity and secrete proteolytic enzymes to kill them [54, 55]. In addition, many studies have provided evidence that plasma factors can play an important role in the host immune response by interacting with hemocytes [16, 27, 56].

Interestingly, in this study, the greatest number of differentially expressed proteins from hemocytes was observed at 24 and 48 hpi (79 and 86, respectively), with a lower number of differentially expressed proteins at 96 hpi (only 28), whereas several plasma proteins were present at different levels throughout the study. According to a previous report on *B*. *siamensis goniomphalos* infected with *O*. *viverrini*, the majority of differentially expressed proteins on the whole snail’s proteome were observed at 28–56 days post infection [29]. It is likely that the changes in the protein expression observed in these times could be linked to the parasite migration through digestive glands where asexual reproduction and parasite localization within the snail host take place. However, our findings could be indicating that the changes observed in the expression of hemocyte proteins could be a direct response against *O*. *viverrini* infection in early infection. Within hours of infection, the miracidium hatches from the egg, penetrates the snail tissue and transforms into the sporocyst within 4 h [5]. In *B*. *glabrata*, the snail’s immune system is directed towards the tegument and ciliated plates shed by the miracidium when it transforms into the sporocyst stage [57], followed by a strong up-regulation of a number of defense-related transcripts in early infection (12–48 hpi), and, a down-regulation at and beyond 4 days post-infection [58, 59]. The results observed in the present study suggest that early stages of the infection are critical for the success or failure of parasite establishment.

A GO analysis was also performed on proteins with significantly different expression patterns, which were classified into functional groups accordingly (Fig 3). The main abundant GO term represented in biological process was the immune system process. Several immune-relevant proteins were identified in this group, such as C-type lectin, galectin-4, heat shock proteins (HSPs), glutathione-s-transferases (GSTs), hemocyanin (HMC), serpin and peroxiredoxin. HMC is significantly expressed in both hemocyte and plasma after infection, although at different times post-infection. The expression of HMC was strongly up-regulated at 24 hpi and particularly at 48 hpi in hemocyte, while, in plasma, it was down-regulated at 48 hpi but up-regulated at 96 hpi. HMC is the main protein component of hemolymph, and is found in both hemocyte and plasma. The function HMC in invertebrate is not only as a respiratory protein via oxygen transport, but it has also been strongly associated with different immunological processes [60–66]. HMC has been shown to participate in multiple roles of the immune response including phenoloxidase activity [61–64], antiviral [65] and antimicrobial activity [66]. In snails, a study on the transcriptional profile of *B*. *glabrata* snails showed that a number of HMC transcripts were up-regulated after *S*. *mansoni* and *Echinostoma paraensei* infection [59]. A previous study on *B*. *siamensis goniomphalos* revealed that the expression of HMC was significantly up-regulated in uninfected snails compared to infected snails [29], however, this study was performed on the head-foot and the body of the snails and not on the cells or tissues involved in the immune response. In our case, hemocytes could be expressing more HMC to fight *O*. *viverrini* infection in the early stages of the infection. Once the infection is cleared, HMC is no longer needed and its expression levels would be expected to decrease. In the case of plasma, the high levels of HMC found at 96 hpi could be related to lysis of hemocytes that would release their contents, although more studies should be done in this regard.

Immunoglobulin superfamily (IgSF) proteins act as antibodies in mammals. While invertebrates do not have antibodies, some invertebrate molecules containing IgSF domain proteins are commonly associated with the immune system as they have the ability to specifically recognize pathogen molecules [26, 67–69]. In our study, 7 and 13 IgSF domain-containing proteins were identified in the hemocytes and plasma libraries, respectively. From these, 1 and 8 proteins had significantly up-regulated expression profiles in hemocytes and plasma, respectively, and the expression of only 1 protein (similar to IgSF DCC subclass member 4 [70]) was down-regulated. These results suggest that the proteins containing an IgSF domain might play a role in *Bithynia* snail defense responses against *O*. *viverrini* infection. In a similar way, lectins are known to play a role in molecule recognition and are involved in both humoral and cellular immune response in molluscs [25, 30]. Recently, a comprehensive study reported different proteins involved in the immune response containing one or two IgSF domains and a multi lectin domain such as fibrinogen-related proteins (FREPs), C-type lectin-related proteins (CREPs) and galectin-related proteins (GREPs) in *B*. *glabrata* [71]. FREPs are characterized as containing a C-terminal fibrinogen domain and one or two IgSF-like domains at the N-terminus, and are involved in *B*. *glabrata* immune responses [26, 72, 73]. CREPs and GREPs consist of one or two IgSF domains followed by a C-type lectin or a galectin domain, respectively [30, 71], which are a family of glycan-binding proteins that are able to recognize and bind to the tegument of pathogens, and are usually up-regulated after infection [31, 74, 75]. In our study, only 6 proteins in the hemocyte library contained a fibrinogen domain, of which 2 had a significantly down-regulated expression pattern in hemocytes, although none of these 6 proteins had an IgSF domain. Moreover, 2 proteins with a significantly regulated expression profile containing a C-type lectin domain (such as a protein similar to galactose-specific lectin nattectin [33] and a protein having low similarity with macrophage manose receptor-1 like protein from *B*. *glabrata*) were identified. Furthermore, we identified a protein containing a galactoside-binding lectin domain that was similar to galectin-4. However, none of the proteins contained an IgSF domain. While we cannot rule out the possibility that these are partial sequences and the IgSF domain could be present, the fact that the expression of all of these lectin domain-containing proteins was down-regulated could indicate that they are not true FREPs, CREPs or GREPs, and, thus, would not be involved in the snail’s immune response against the parasite, or alternatively might be the result of parasite-induced immunosuppression. This is in disagreement with previously reported literature on other snails acting as trematode intermediate hosts such as *B*. *glabrata*. However, it is noteworthy that while *B*. *glabrata* is a pulmonate snail, *B*. *siamensis goniomphalos* is a prosobranch. The two groups also show some physiological differences according to evolutionary distance that could affect the different responses of FREPs, CREPs and GREPs against parasitic infections. Despite the fact that several lectin families have been identified in other prosobranchs such as *Littorina littorea* [76], there is no information published regarding the expression of these molecules following parasite infection. In order to unravel the functions of *B*. *siamensis goniomphalos* lectins in snail-fluke interactions, further studies are needed. In this sense, the snail’s genome sequence will be of great interest.

Other proteins defined as “stress-related responses”, including heat shock proteins (HSPs), were significantly differentially expressed. Three different HSPs (HSP-70, HSP-83 and HSP-90) were detected in the hemocytes of infected snails, and their expression was down-regulated at 24 and 48 hpi, whereas only HSP-70 was found in plasma, and its expression was up-regulated at 24 and 48 hpi. It has been previously reported that the expression levels of HSP70 were down-regulated in *B*. *glabrata* hemocytes after incubation with excretory/secretory products (ES) from *S*. *mansoni* [77], which was linked to a parasite strategy to manipulate the snail’s immune response. Conversely, other studies have described an up-regulation of the expression levels of this protein as a result of parasite infection [34, 36, 78], so the role of these proteins in the snail’s defense against pathogens is not entirely clear.

In addition, several proteins corresponding to enzymatic activity were characterized including glutathione-s-transferases (GSTs), fructose-bisphosphate aldolase, serpin and peroxiredoxin. Detoxifying enzymes such as GSTs and peroxiredoxins play an important role in removing harmful free radicals or cytotoxic compounds to protect the snail host during infection and the ensuing immune response, and their regulation could be linked to a survival strategy against infection [31, 59]. We detected significant down-regulation of the expression of GST whereas the expression of peroxiredoxins was up-regulated in plasma. In *B*. *glabrata* snails, it has been previously shown that the differential capability of host defense responses in susceptible and resistant snails could have different effects on enzyme activity due to the differences in the levels of these enzymes in each strain [31, 79, 80]. Serpin is a serine protease inhibitor that was only found expressed in plasma with an up-regulated expression profile at 24 and 48 hpi. This protein is strongly implicated in the innate immune response, and is likely to function by inactivating and clearing proteases such as those present in the ES of the parasite produced during invasion of the host [59, 74, 81]. The expression levels of several actin-related proteins (e.g. actin, filamin-4, tubulin, myosin and twitchin) were down-regulated in hemocytes and up-regulated in plasma. These proteins are involved in various types of cell motility and cell-cell interactions. Recent studies have revealed that actin-related genes and proteins were consistently up-regulated in infected *Bithynia* snails, and it was hypothesized that these proteins could be associated with hemocyte chemotaxis and with the promotion of phagocytosis and encapsulation against parasite infection [29, 36]. Moreover, we found fructose-1,6-bisphosphate aldolase, a glycolytic enzyme that can interact with cytoskeletal proteins and could play a role in the motility or adhesion of hemocytes. Fructose-1,6-bisphosphate aldolase has been identified in hemocytes of *B*. *glabrata* and has been shown to regulate hemocyte migration, implicating this protein in pathogen killing via cytotoxic reactivity and phagocytosis [28].

In this study, we employed the state-of-the-art proteomic technique SWATH-MS to identify and quantify the changes in proteins from hemocytes and plasma of *B*. *siamensis goniomphalos* following *O*. *viverrini* infection. We revealed several proteins potentially involved in the immune response of the snails that could play a critical role in the snail-trematode relationship, although future functional studies are needed to clarify the role of these proteins and better understand the immune response of *Bithynia* snails to *O*. *viverrini*.

## Supporting Information

S1 Table List of protein expression from hemocytes assay library. The table includes the following columns: protein identifier based on the transcriptome previously published [35], peptide sequence identified, the precursor charge, the fragment ion, product charge, the condition (time point studied), bioreplicate (biological replicate in which the peptide was identified), and area (intensity measurement used for quantification purposes).
(XLSX)

S2 Table List of protein expression from plasma assay library. The table includes the following columns: protein identifier based on the transcriptome previously published [35], peptide sequence identified, the precursor charge, the fragment ion, product charge, the condition (time point studied), bioreplicate (biological replicate in which the peptide was identified), and area (intensity measurement used for quantification purposes).
(XLSX)

S3 Table Statistical information on the significantly differentially expressed proteins from hemocytes. MSstats output table including the protein identifier, label (group comparison), log_2_ fold change, standard error, T value, degree of freedom, raw p-value and adjusted p-value based on Benjamini and Hochberg method to collect multiple testing issue and further control false discovery rate (adj.pvalue).
(XLSX)

S4 Table Statistical information on the significantly differentially expressed proteins from plasma. MSstats output table including the protein identifier, label (group comparison), log_2_ fold change, standard error, T value, degree of freedom, raw p-value and adjusted p-value based on Benjamini and Hochberg method to collect multiple testing issue and further control false discovery rate (adj.pvalue).
(XLSX)

S5 Table Log_2_ fold-change of significantly differentially expressed proteins from hemocytes. The table includes also the blast name results (description) and gene ontology categories for each protein. Control values have been normalized to 0 for comparison to the other time-points and a '-' indicates that the fold-change was non-significant and therefore were omitted from the supplementary table.
(XLSX)

S6 Table Log_2_ fold-change of significantly differentially expressed proteins from plasma. The table includes also the blast name results (description) and gene ontology categories for each protein. Control values have been normalized to 0 for comparison to the other time-points and a '-' indicates that the fold-change was non-significant and therefore were omitted from the supplementary table.
(XLSX)

S1 Fig Agarose gel showing the PCR products from infected and uninfected snails. Snails were screened for trematode infection by PCR designed to amplify the ITS regions. Only a representation of the 1,200 (900 infected and 300 uninfected) snails is shown. Lanes 1–10 shows the amplified ITS region of *Opisthorchis viverrini* from infected snails, while lanes 11–20 shows the results of uninfected snails. (+) positive control; (-) negative control.
(JPG)

S2 Fig Venn diagram of SWATH-MS identified proteins. Venn diagram showing the overlap of differentially expressed proteins at the different time points in hemocytes (A) and plasma (B).
(TIFF)
