# Supplementary figures and images for: Differential Protein Expression in the Hemolymph of Bithynia siamensis goniomphalos Infected with Opisthorchis viverrini
Source: PLoS Negl Trop Dis. 2016 Nov 28;10(11):e0005104. doi: 10.1371/journal.pntd.0005104 (PMC5125571; doi:10.1371/journal.pntd.0005104)

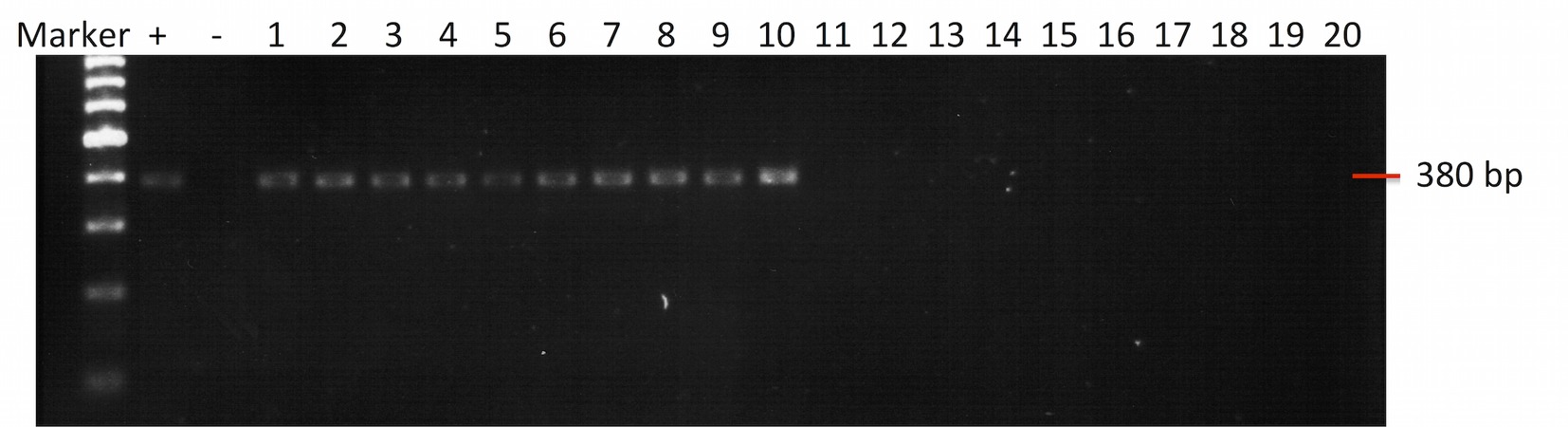

Supplement: S1 Fig — Snails were screened for trematode infection by PCR designed to amplify the ITS regions. Only a representation of the 1,200 (900 infected and 300 uninfected) snails is shown. Lanes 1–10 shows the amplified ITS region of Opisthorchis viverrini from infected snails, while lanes 11–20 shows the results of uninfected snails. (+) positive control; (-) negative control. (JPG) [file pntd.0005104.s007.jpg]

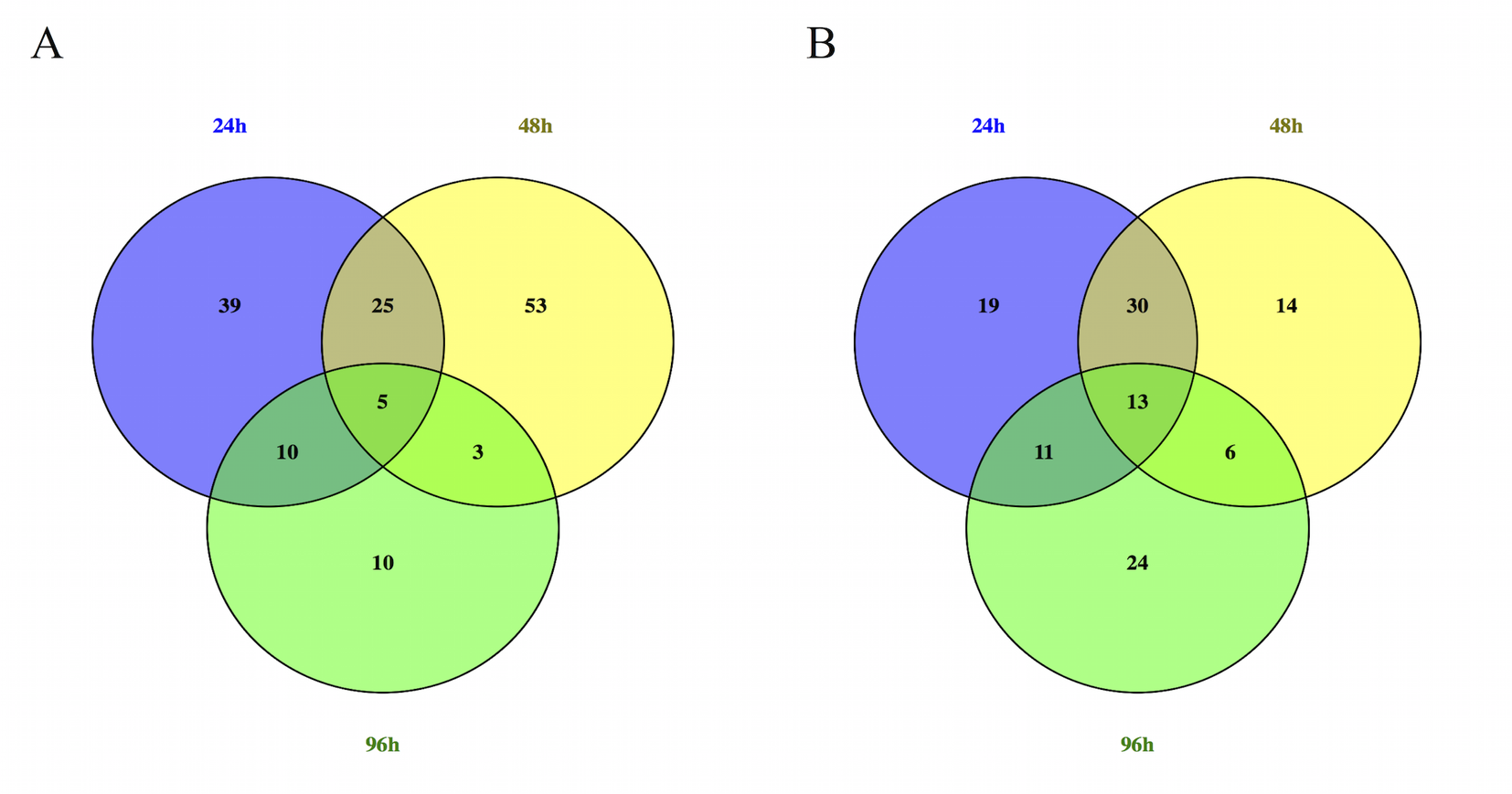

Supplement: S2 Fig — Venn diagram showing the overlap of differentially expressed proteins at the different time points in hemocytes (A) and plasma (B). (TIFF) [file pntd.0005104.s008.tiff]
